# Comparison of manual and semi-automatic registration in augmented reality image-guided liver surgery: a clinical feasibility study

**DOI:** 10.1007/s00464-020-07807-x

**Published:** 2020-08-11

**Authors:** C. Schneider, S. Thompson, J. Totz, Y. Song, M. Allam, M. H. Sodergren, A. E. Desjardins, D. Barratt, S. Ourselin, K. Gurusamy, D. Stoyanov, M. J. Clarkson, D. J. Hawkes, B. R. Davidson

**Affiliations:** 1 Division of Surgery & Interventional Science, Royal Free Campus, University College London, Pond Street, London, NW3 2QG UK; 2 Wellcome / EPSRC Centre for Surgical and Interventional Sciences (WEISS), University College London, London, UK; 3 Centre for Medical Image Computing (CMIC), University College London, London, UK; 4 Department of Hepatopancreatobiliary and Liver Transplant Surgery, Royal Free Hospital, London, UK; 5 Department of Medical Physics and Bioengineering, University College London, London, UK; 6 Department of Computer Science, University College London, London, UK

**Keywords:** Augmented reality, Image-guided surgery, Laparoscopic liver surgery, Computer-assisted surgery, Stereoscopic surface reconstruction, Semi-automatic registration

## Abstract

**Background:**

The laparoscopic approach to liver resection may reduce morbidity and hospital stay. However, uptake has been slow due to concerns about patient safety and oncological radicality. Image guidance systems may improve patient safety by enabling 3D visualisation of critical intra- and extrahepatic structures. Current systems suffer from non-intuitive visualisation and a complicated setup process. A novel image guidance system (SmartLiver), offering augmented reality visualisation and semi-automatic registration has been developed to address these issues. A clinical feasibility study evaluated the performance and usability of SmartLiver with either manual or semi-automatic registration.

**Methods:**

Intraoperative image guidance data were recorded and analysed in patients undergoing laparoscopic liver resection or cancer staging. Stereoscopic surface reconstruction and iterative closest point matching facilitated semi-automatic registration. The primary endpoint was defined as successful registration as determined by the operating surgeon. Secondary endpoints were system usability as assessed by a surgeon questionnaire and comparison of manual vs. semi-automatic registration accuracy. Since SmartLiver is still in development no attempt was made to evaluate its impact on perioperative outcomes.

**Results:**

The primary endpoint was achieved in 16 out of 18 patients. Initially semi-automatic registration failed because the IGS could not distinguish the liver surface from surrounding structures. Implementation of a deep learning algorithm enabled the IGS to overcome this issue and facilitate semi-automatic registration. Mean registration accuracy was 10.9 ± 4.2 mm (manual) vs. 13.9 ± 4.4 mm (semi-automatic) (Mean difference − 3 mm; *p* = 0.158). Surgeon feedback was positive about IGS handling and improved intraoperative orientation but also highlighted the need for a simpler setup process and better integration with laparoscopic ultrasound.

**Conclusion:**

The technical feasibility of using SmartLiver intraoperatively has been demonstrated. With further improvements semi-automatic registration may enhance user friendliness and workflow of SmartLiver. Manual and semi-automatic registration accuracy were comparable but evaluation on a larger patient cohort is required to confirm these findings.

Laparoscopic liver resection (LLR) reduces pain and complications resulting in shorter hospital stay with comparable oncological outcomes to open liver resection [1–4]. Uptake of the laparoscopic approach has been slow [3, 5] but is progressing [4] with most HPB centres carrying out at least minor liver resections laparoscopically whilst only a few centres perform major hepatectomies or complex liver resections (e.g. superior-posterior segments), laparoscopically [4, 5]. Expansion of laparoscopic liver surgery is slowed by inherent limitations to depth perception, tactile feedback and field of view which are compounded by the livers varied and complex anatomy [6, 7]. These limitations have given rise to concern over controlling bleeding and ensuring adequate oncological clearance [3–5, 8–10].

Navigated image guidance systems (IGS) have been shown to improve outcomes in neurosurgery [11–13], and have also been applied to LLR with the aim of enhancing intraoperative orientation and to improve safety [7, 14–16]. IGS allow surgeons to view structures, such as tumours and blood vessels, that can be seen on preoperative scans but that are not visible with a laparoscopic camera [16, 17].

Laparoscopic ultrasound, an alternative technique for operative imaging is limited by its two-dimensionality and poor contrast between tumours and normal liver [7, 18–20]. Video-based IGS using augmented reality (AR) can superimposes a 3D liver model directly onto the laparoscopic screen [21, 22]. Generally application of these systems requires three key steps; the creation of a personalised 3D liver model from a preoperative CT or MRI scan, intraoperative image registration and tracking of the laparoscope to guide the image overlay.

Two commercial IGS designed for open liver surgery [23, 24], have been adapted for LLR with studies demonstrating comparable accuracy to open surgery [7, 22]. These systems however are limited by the need for separate screens to demonstrate image guidance [7] and the use of manual registration [7, 22] which is a source of errors and delay to the intraoperative workflow [25]. To address these issues an IGS is being developed with capabilities for AR and semi-automatic registration [26, 27]. These features may improve the performance and usability of navigated image guidance. The current study tests the feasibility of using the new IGS, Smart Liver, in a clinical setting and is the first study to compare manual with semi-automatic registration [28].

## Methods

A novel image-guided surgery system (SmartLiver) was designed for use in LLR through a programme of basic research and clinical development commissioned by the Wellcome Trust in partnership with the Department of Health (UK) [21, 26, 27, 29].

### System description

**Fig. 1:**
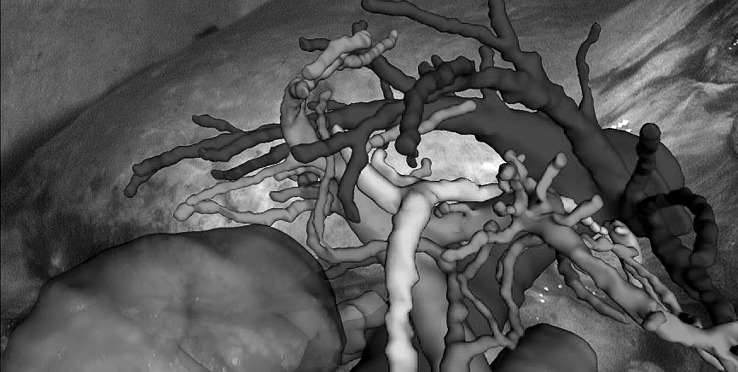
Augmented reality visualisation of a 3D liver model overlayed onto the laparoscopic view. The liver surface outline (arrows) is not displayed to allow a clearer view of blood vessels and bile ducts (hepatic veins—blue; portal veins—purple; arteries—red, bile ducts & gallbladder—green). NB: The text on top of the image will be removed for the revised version of the manuscript (Color figure online)

**Fig. 2:**
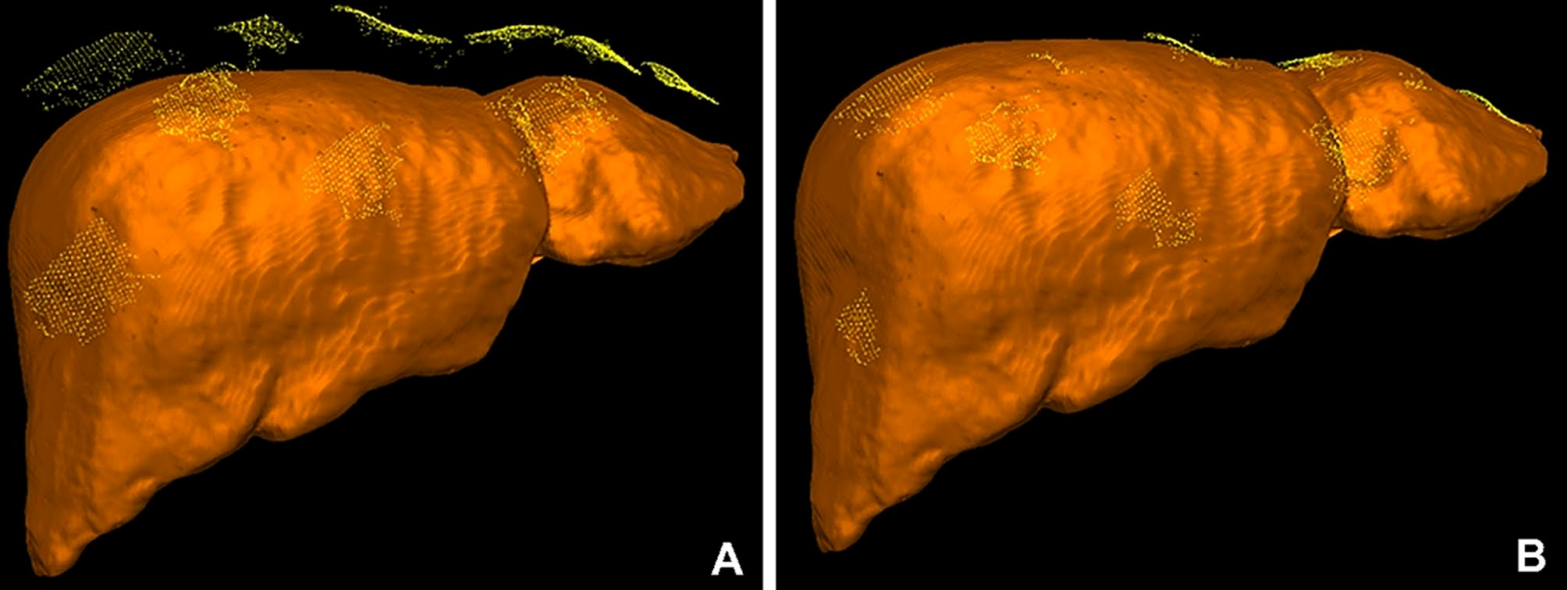
**A** Several patches of point clouds (yellow dots) represent the shape of the liver surface. The un-registered (non-aligned) position of the 3D model can be seen as a brown liver shape below the patches. **B** Following iterative closest point matching, the semi-automatic registration algorithm has positioned the 3D liver model optimally to reflect the intraoperative anatomy

The 3D models used for AR visualisation (Fig. 1) were produced by Visible Patient™ (Strasbourg, France). In brief, 3D models were constructed from the contrast enhanced CT carried out as part of routine investigations for patients with suspected hepato-pancreato-biliary malignancy. The Polaris Spectra™ system (NDI Medical, Waterloo, Canada) was employed for optical tracking of the laparoscope [26]. SmartLiver is being developed to function with either manual or semi-automatic registration. Manual registration is controlled by a touch screen monitor or mouse which enables manipulation of the 3D model into an anatomically appropriate position. Semi-automatic registration is facilitated by a computer vision technique called stereoscopic surface reconstruction which enables the acquisition of the biometrical liver surface features (dense surface reconstruction) that are subsequently represented as 3D points cloud. Stereoscopic surface reconstruction functions by triangulating the right and left video channel of a 3D laparoscope (IMAGE 1S—TIPCAM, KARL STORZ™, Tuttlingen, Germany) and therefore this technique cannot be applied to standard monocular laparoscopes [30]. Using the iterative closest point matching method, corresponding 3D points cloud from patient liver and 3D liver model are aligned to complete the registration [26, 27] (Fig. 2). Iterative closest point matching is initialised by manually positioning the 3D model in proximity to the patient liver. Following liver mobilisation the registration process was usually repeated to adjust for liver position changes. For in-depth details on SmartLiver technology please see [21, 26, 27].

### Workflow in theatre

**Fig. 3:**
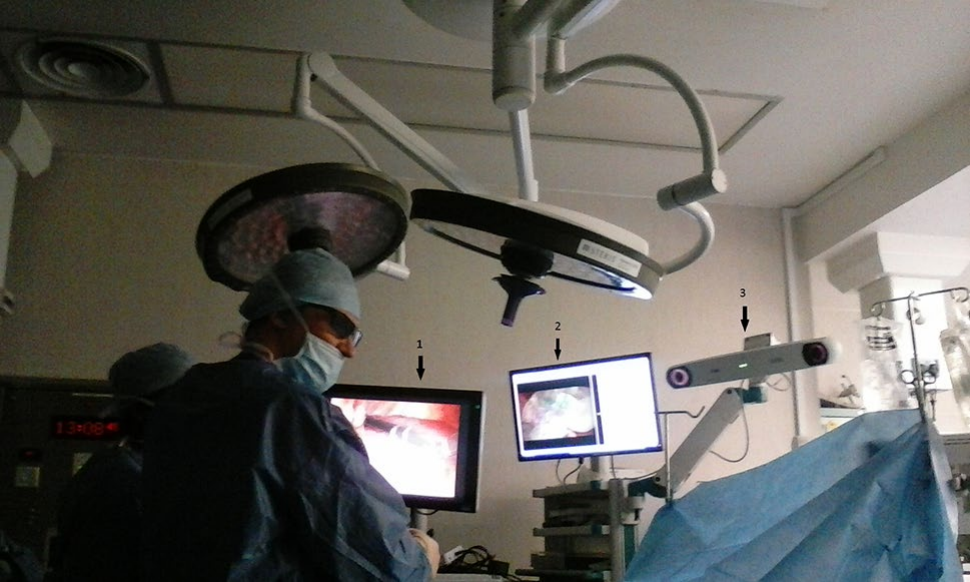
The surgeon uses a standard laparoscopic screen (1) whilst the research team uses a separate screen (2) for calibration, registration and data capture. In the later phase of the study the surgeon is allowed to visualise the AR view through this screen. The optical tracking camera (3) is attached to an adjustable arm

Two separate stacks are used. One 3D laparoscope stack with its own screen and a second stack that contains all components of SmartLiver including a flexible arm for the optical tracking sensor which is positioned at the head end of the operating table to obtain an unobstructed line of sight of the laparoscope (Fig. 3). Finally the laparoscope is calibrated according to the Zhang [31] chequerboard method or in later cases according to the novel “cross-hair” method that was developed by our team [32].

### Patients

The study was approved by the local research ethics committee (Reference: 14/LO/1264 & 10/HO720/87) and registered with ISRCTN (ID: 77923416). Written consent was obtained from recruited patients. Because the accuracy of SmartLiver was unknown at the outset, the ethics approval did not permit surgeons to use the IGS to adjust operative strategy. For this reason this study did not evaluate SmartLiver’s impact on surgical outcomes but rather the feasibility of intraoperative use.

Patients who were 18 years or older undergoing staging laparoscopy or LLR were eligible for recruitment. Demographic information and perioperative data were recorded for all patients. In addition to these the conversion rate, need for perioperative blood transfusion, postoperative complications (Clavien-Dindo grade), resection margin status and length of hospital stay were recorded for LLR patients [33]. Clinical evaluation was supervised by an HPB surgeon with over 15 years experience in LLR.

### Task description and endpoints

The aim of the study was to assess the feasibility of using SmartLiver for image guidance in laparoscopic liver surgery and to compare navigation accuracy between manual and semi-automatic registration. The primary endpoint was defined as successful registration as determined by the operating surgeon who had reviewed the preoperative imaging and was judged on whether the 3D model maintained an anatomically appropriate and stable position during surgery. Failure of semi-automatic registration resulted in an error message.

**Fig. 4:**
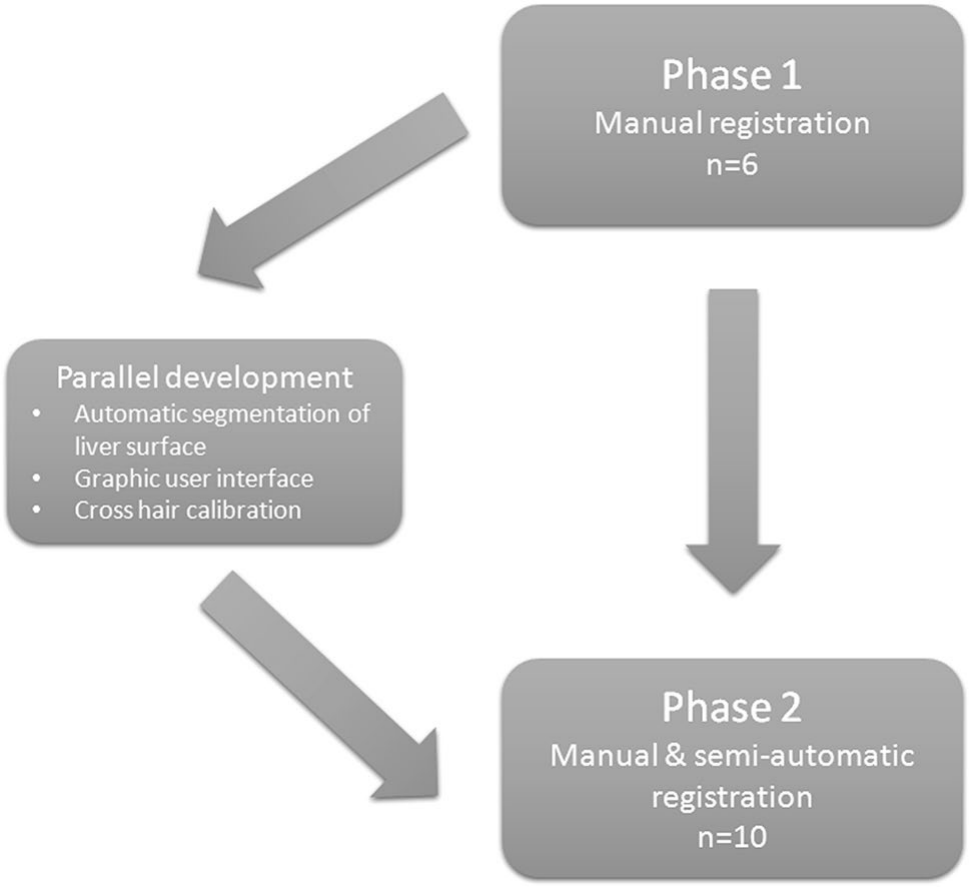
Study structure. In phase one, registration was retrospective whereas intraoperative registration was carried out in phase two. The data from phase one were used to drive improvements to SmartLiver which were implemented in phase two

Secondary endpoints were system usability and comparison of the accuracy of manual and semi-automatic registration. Data for registration were obtained by recording the liver surface from different laparoscope angles. Registration was carried out by a technical developer. Because of ongoing system development intraoperative registration was found to be very time consuming. Therefore postoperative registration, based on intraoperatively recorded data, was performed in phase one. This approach allowed us to obtain the data required to improve workflow and system functionality, in a more time efficient manner. In the second phase, registration was carried out intraoperatively and additional surface data were acquired to facilitate semi-automatic registration (Fig. 4). In cases where intraoperative registration failed, retrospective registration was performed.

### Usability evaluation

Usability was assessed by a structured Likert scale survey which was completed by the primary surgeon, postoperatively. The survey also encouraged comments in a free text section. In the first study phase surgeons could not view the AR visualisation because it was carried out retrospectively. Therefore survey questions were changed in the second phase to include feedback on how well AR visualisation reflected the anatomical situation. To aid in this assessment surgeons compared the congruence of external liver landmarks (e.g. extrahepatic bile duct, left liver margin, umbilical fissure) between laparoscopic display and registered 3D model.

### Comparison of manual and semi-automatic registration

**Fig. 5:**
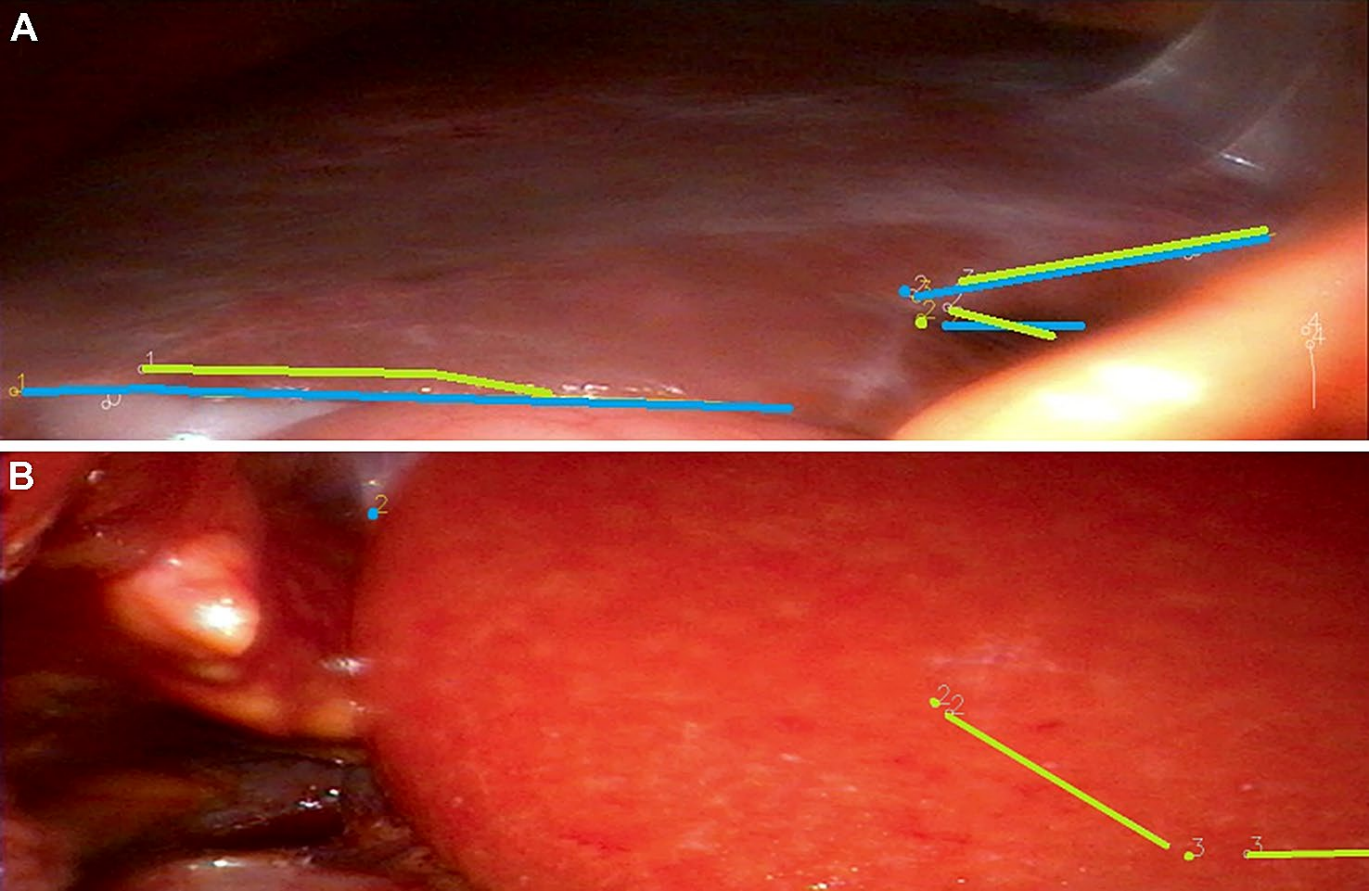
**A** A registration with a low error results in relative proximity of patient anatomy (blue landmarks) and 3D model anatomy (green landmarks). **B** In contrast to this a registration with a substantial error results in long distance between the corresponding landmarks. NB: The landmarks have been highlighted to enhance visibility. Landmark 3 is outside the visible area of the screen

Due to the lack of standardised methods for assessing IGS navigation accuracy [34], our group previously proposed a landmark based method which was also employed here [21, 26]. In brief, distances between corresponding anatomical landmarks on laparoscopic images and the 3D liver model are measured and compared (Fig. 5). Essentially, an increase in the measured distance results in an increased registration error (i.e. decreased accuracy). Distance measurements are possible because the registration process between the 3D model, its inherent volumetric data and laparoscopic images creates fixed reference points akin to a 3D coordinate system. These reference points subsequently enable extrapolation of distance measurements from laparoscopic images [26]. Common liver landmarks used for accuracy calculation were the left lateral margin, lower margin, falciform ligament and umbilical notch. Occasionally patients had unique anatomical features such as liver indentations, scarring or superficial liver cysts that could also be used as landmarks. To provide an estimate of the optimal target registration error (TRE), the root mean square value of all individual distance errors (i.e. distances) is calculated across multiple video frames and stated in millimetre root mean square (abbreviated to mm). Root mean square is defined as the square root of the mean square which is the arithmetic mean of the squares of all distance errors. Shapiro-Wilks testing was conducted to check for normal distribution of data. TRE values for groups of patients are stated as mean ± standard deviation (SD). To test accuracy results for statistically significant differences between groups, independent and paired t-testing was used as appropriate. For in-depth details on accuracy evaluation please see [21, 26].

## Results

### Patient characteristics and data acquisition

Eighteen patients underwent image-guided surgery, of which 7 were scheduled for LLR and 11 for staging laparoscopy. The gender ratio was 8 women to 10 men and the median age was 61.5 years (range 38–87). All staging laparoscopies were done as day case surgery. The only complication was urinary retention in one patient who was discharged on the 4th postoperative day. LLR patient characteristics are summarised in Table 1. Median operating time was 150 min (range 75–330 min). All patients had clear resection margins on histopathological evaluation. There was one significant (Clavien-Dindo ≥ 3) postoperative complication (grade 4) in a patient who required re-intubation on postoperative day 2 for respiratory failure. Including patients converted to open surgery the median length of hospital stay was 6.5 days (range 3–14). None of the patients had significant blood loss.

**Table 1:** Patient characteristics for laparoscopic liver resection

Patient ID	Pathology	Anaesthetic time (min)	Conversion to open	Liver segment	Type of resection	Lesion size (largest in mm)	LOS
LR01	Adenoma	120	n	5 & 6	Wedge resection	60	6
LR02	CRLM	150	n	5 & 6	Wedge resection	25	3
LR03	CRLM	270	y	3 & 4	Left hepatectomy	84	8
LR04	HCC	150	y	4a & 4b	Left hepatectomy	60	14
LR05	CRLM	150	n	2	Left lateral sectionectomy	10	5
LR06	CRLM	330	n	4b & 5	Wedge resection	60	9
LR07	BRC	75	n	4b	Wedge resection	18	4

*CCA* cholangiocarcinoma, *CRLM* colorectal cancer liver, *BRC* breast cancer liver metastasis, *HCC* hepatocellular carcinoma, *LOS* length of hospital stay

### Success of registration

Registration failed in two patients. In one patient a software failure of the graphic user interface prevented registration. In the other patient tracking markers became dislodged during surgery which caused tracking issues. Therefore 16 patients had successful registration and were suitable for further analysis.

### Usability evaluation

Preoperative setup took 20–35 min. Because it was carried out before anaesthetic induction it did not impact on overall operating time. Intraoperative setup took approximately 10–15 min. Following the introduction of the crosshair calibration method [32] intraoperative setup time decreased to approximately 5–10 min.

### Usability assessment

Feedback data were available from 10 individual surgeons carrying out 16 cases. Feedback from phase one where AR was demonstrated after the operative procedure is given in the first section of Table 2. Free text comments expressed the desire for a simplified setup process and a more compact system. Feedback from phase two where AR visualisation was presented throughout surgery is given in the second section of Table 2. Free text comments requested a better way of combining SmartLiver with laparoscopic ultrasound and increasing setup speed. One surgeon indicated that the camera optics made it difficult to reach certain angles (Table 2).

**Table 2:** Summary of surgeon feedback on a Likert scale of 1–5

Question	Median
*Phase 1 (n* = *6)*	
Did the system impair handling of the laparoscopic camera?	4
Did the system impair handling of the laparoscopic instruments?	5
How easy was the system to setup?	3
Did you feel that the equipment setup caused delay in completing the surgical procedure?	3
Did the system setup impair your line of view of the patient?	5
Did the system setup impair your line of view of the laparoscopic monitor?	5
Were you overall satisfied with the positioning of the system within the theatre environment?	4
*Phase 2 (n* = *10)*	
Image guidance system use improved orientation of the laparoscope within the body:	4
The overlay as displayed appeared to be in an anatomical correct position:	3
Overlay position was consistent when viewed from different angles:	4
Overlay position did not change during the procedure, even when switched on/off	4
Image guidance system use resulted in better detection of extrahepatic vascular structures	4
Image guidance system use enabled better interpretation of ultrasound images (if US used)	2
Mental integration of the image overlay into the operative workflow is intuitive	4
The time required for setup of the image guidance system does not interrupt the surgical workflow	1

1 (very unsatisfied), 5 (very satisfied). Ten different surgeons provided feedback on 16 operations

### SmartLiver upgrades before phase two

User handling was improved by the implementation of a graphic user interface with touchscreen controls. Laparoscope calibration was simplified and rendered less time consuming by replacing the chequerboard with the crosshair calibration method [32].

Despite encouraging results from pre-clinical studies [26], stereoscopic surface reconstruction was initially unsuccessful in patients. An error analysis indicated that the stereoscopic surface reconstruction algorithm was unable to discriminate between surface points from the liver and surrounding structures (e.g. diaphragm). Based on data from phase one, a convolutional neural network, was trained to perform automatic segmentation (i.e. recognition) of the liver surface [35]. Following integration into SmartLiver, this algorithm subsequently enabled successful stereoscopic surface reconstruction and semi-automatic registration in study phase two.

### Comparison of manual and semi-automatic registration

In phase one, retrospective manual registration was performed in 6 patients. In phase two, both manual and semi-automatic registration were performed in 10 patients. Accuracy for manual registration in phase one was 15.8 ± 14.2 mm. In phase two manual registration accuracy improved to 10.9 ± 4.2 mm. This improvement did not reach statistical significance with a mean difference of 4.9 mm (− 1.1 to 10.9 mm 95% CI; p = 0.104). Semi-automatic registration accuracy in phase two was 13.9 ± 4.4 mm which was not statistically significant different compared to manual registration with a mean difference of − 3 mm (− 7.4 to 1.4 mm 95% CI; p = 0.158) (Table 3).

**Table 3:** Accuracy values in phase one and two of the study

Patient ID	TRE manual	TRE semi-automatic
*Phase 1*		
LR01	15.4	n.a
LR02	22.7	n.a
LS03	24.6	n.a
LS04	5.9	n.a
LS06	16.1	n.a
LS07	10.0	n.a
Mean ± SD	15.8 ± 7.2	n.a
*Phase 2*		
LR04	9.2	10.4
LS05	10.6	8.7
LS08	17.5	16.8
LR06	9.8	9.8
LR07	12.5	20.8
LS09	16.1	11.6
LS10	9.6	11.1
LS11	12.9	16.8
LS15	2.8	13.0
LR08	8.0	19.9
Mean ± SD	10.9 ± 4.2	13.9 ± 4.4

Accuracy is stated as mm RMS

*SD* standard deviation, *TRE* target registration error

## Discussion

This study has described the development and current performance of the SmartLiver IGS. Focus was on feasibility as opposed to clinical impact because at the outset, navigation accuracy was unknown and therefore no ethical approval was sought to use SmartLiver to adjust surgical strategy. Evaluation was carried out on 18 patients undergoing either LLR or staging laparoscopy. There were no patient safety incidents associated with the use of SmartLiver and perioperative outcomes for patients undergoing LLR were similar to previous reports [4, 36, 37]. Although IGS are widely used in neurosurgery, orthopaedic surgery and otolaryngology, implementation in LLR has been slow and difficult [38]. Major challenges are the lack of fixed bony landmarks, paucity of liver surface features, organ motion secondary to diaphragmatic and cardiac movement as well as soft tissue deformation due pneumoperitoneum and surgical manipulation [7, 38, 39].

The experimental work leading up to this study demonstrated that liver motion and deformation, contribute approximately 7.5 mm to the TRE of SmartLiver [26, 39, 40]. To achieve a greater level of accuracy requires a deformable 3D model that can adjust its shape and position to reflect intraoperative changes [26]. The research community has attempted to develop deformable 3D models with varying degrees of success. Because modelling of soft tissue deformation is exceedingly complex and computationally expensive, this technology has not yet reached sufficient maturity for clinical studies [41, 42].

The primary endpoint of successful registration as assessed by the operating surgeon was achieved in 16 out of 18 patients. Success was indicated by the 3D model maintaining an anatomically appropriate and stable position. It has been previously reported that liver mobilisation results in significant positional shift of landmarks and therefore necessitates repeat registration [43]. Although not formally quantified, this was also the case in the current study. Hypothetically image guidance should be most beneficial during dissection at the liver hilum and parenchymal transection since the exact position of intrahepatic structures and tumours are crucial during these steps. Liver mobilisation is a standardised process and therefore registration and image guidance may be less important at this stage. Although re-registration issues affect all current IGS, we strongly believe that semi-automatic registration renders this process less cumbersome.

During the first study phase, registration was carried out postoperatively which meant that surgeons could not evaluate the quality AR visualisation. Feedback about equipment handling was positive whereas negative feedback mainly centred on the complexity of the intraoperative setup (e.g. tracker installation) and its impact on surgical workflow. To simplify the setup process, our group developed the crosshair calibration method and a graphic user interface [29]. In the second study phase, AR visualisation was evaluated intraoperatively. Positive feedback points were that SmartLiver improved intraoperative orientation, aided in the detection of extrahepatic structures and was consistent in the way it displayed anatomy (Table 2). Feedback about the combination of SmartLiver with laparoscopic ultrasound was less favourable, because viewing both, the AR and ultrasound -screen simultaneously was challenging. Our group previously demonstrated how SmartLiver can effectively integrate ultrasound images into AR [44]. This approach however requires electromagnetically tracked ultrasound, which was not ethically approved for this study. The anatomical precision of the overlay also received negative feedback which probably reflects the fact that there were obvious discrepancies between 3D model position and corresponding liver sections in 4 patients with a higher than average TRE. To improve anatomical precision, efforts were increased to improve manual registration accuracy. In contrast to our groups experience from porcine studies [26], it was observed that, in patients stereoscopic surface reconstruction may misalign different anatomical regions (e.g. diaphragm with liver), if they have a similar surface structure. Hypothetically the coarser, more lobulated surface of the porcine liver may be more amenable to stereoscopic surface reconstruction because it contains more features to distinguish it from surrounding structures. As demonstrated on this data set, stereoscopic surface reconstruction for the purpose of semi-automatic registration of the human liver is feasible if the liver surface is automatically segmented prior to registration [35, 45]. To the best of our knowledge this is the first clinical study to compare accuracy of manual and semi-automatic registration in a group of patients. Although the accuracy for manual registration was better than for semi-automatic registration, this did not reach statistical significance. Accuracy of manual registration is comparable to that from other groups previously published in the literature [22, 46, 47]. Various methodologies for accuracy evaluation have been proposed over time which makes direct comparison between different IGS challenging [34]. Generally the best published accuracies for video-based IGS are in the range of 10 mm and thus at the current state of the art, any IGS should perhaps be considered as an orientation aid rather than a precise navigation tool [18, 23, 24]. The utility for visualising intrahepatic structures depended mainly on the quality of the registration. In patients where accuracy ≤ 10 mm was achieved, it was feasible to approximate the position of sectoral branches (e.g. right anterior sector) and major hepatic vein branches. To maximise the potential of IGS it is important to enable smooth integration into the surgical workflow. The main benefit of AR is the intuitive use of image guidance information by obviating the need for two separate screens, therefore reducing the potential for associated errors [14]. Pending further validation, semi-automatic registration could improve user friendliness and render accuracy less operator dependent compared to manual registration [48]. Indeed, time efficiency and operator dependence may be crucial advantages of IGS compared to laparoscopic ultrasound. AR visualisation can be switched on and off within seconds whereas ultrasound requires insertion of a laparoscopic probe and manual scanning of the liver surface. Precise use of laparoscopic ultrasound is heavily operator dependent and has a steep learning curve [49, 50], whereas surgeon feedback indicates that SmartLiver’s AR is easy to mentally integrate.

Although the results from this study have demonstrated the technical feasibility of using SmartLiver intraoperatively, there are some limitations that have to be taken into account. To confirm that the accuracy of semi-automatic registration is non-inferior to manual registration, validation on a larger patient cohort is required. In the second study phase manual registration accuracy was comparable to that of other systems [22, 46, 47]. Since this is the first clinical report on semi-automatic registration in a clinical series, there are no published data to compare our results to.

It is often criticised that liver surface features may be an inadequate representation of intrahepatic anatomy. Our group and others however recently demonstrated that liver surface landmarks have a good correlation with the anatomical location of intrahepatic structures (e.g. blood vessels) [21, 51]. Simultaneous localisation and mapping (SLAM) and 3D pose estimation are alternative approaches to image guidance that do not require tracking and therefore may reduce complexity of IGS setup and handling. It remains to be seen however if these technologies can be successfully applied to an intraoperative, clinical setting [15, 52]. Other groups have demonstrated the feasibility of image-guided laparoscopic liver ablation [19, 53]. Hypothetically, SmartLiver has the potential to provide image guidance for laparoscopic liver ablation as well but at present further evaluation is required to verify this. In principal IGS can be applied to robotic assisted surgery [54] without requiring significant alterations. Again our group has not explored this option yet because the main focus is on improving SmartLiver’s performance for LLR first.

In summary, this article has described the clinical development and usability a novel IGS for laparoscopic surgery. For the first time, accuracy metrics for manual and semi-automatic registration have been compared in a clinical series. The next stage of system development will focus on improving SmartLiver’s setup process and to explore alternative methods of semi-automatic registration.

## Abbreviations

3D: Three-dimensional
AR: Augmented reality
CI: Confidence interval
CT: Computer tomography
IGS: Image guidance system
LLR: Laparoscopic liver resection
MRI: Magnetic resonance imaging
TRE: Target registration error
